# The Hospital Problem

**Published:** 1890-03-08

**Authors:** Henry C. Burdett

**Affiliations:** Deputy Chairman of The Hospitals Association


### THE HOSPITAL PROBLEM.

BY MR. HENRY C. BURDETT, Deputy Chairman of The Hospitals Association.

### I.—HOSPITAL INCOME AND EXPENDITURE.

THE subject matter of this and the two articles which are to follow it, has been prepared with a view to helping those engaged in the administration of hospitals and kindred charities.

### HOSPITAL INCOME.

I have analysed the income returns of 159 London provincial, Scotch, and Irish hospitals, and I find the total income amounts to £1,202,462. Of these, 27, having medical schools attached, possess an income of £551,159; 80 general hospitals have collectively an income of £377,384; whilst the income of the remaining 52 special hospitals amounts to £273,886. It will be seen that, although the special hospitals amount to one-third of the whole number of institutions dealt with, they only have an income of rather more than a quarter of a million, or one-fifth of the whole. It would be wearisome and confusing to attempt to deal in detail with these figures, but some instructive facts are deducible from an analysis of the various sources of income and the percentage which each bears to the total.

Thus the general hospitals of London are far behind the special hospitals of the metropolis, and all the other hospitals throughout the country, in the matter of annual subscriptions. Only 12 per cent. of their income is derived from this source, whereas the London special hospitals get 20 per cent. from subscriptions, and in the provinces, Scotland, and Ireland nearly 30 per cent. It follows either that residents in London take relatively small personal interest in the hospitals, or that the managers have failed to appreciate the importance of annual subscriptions, and to devote sufficient effort to induce a large number of persons to add their names to the list of annual subscribers.

Donations represent about 22½ per cent. of the income of general hospitals, the proportion being 21½ per cent. in London, whilst the amount the special hospitals derive from this source represents 27·4 per cent. of the whole. It would be interesting to know how much money has been expended by the special hospitals in order to bring in this extra 6 per cent. from this source.

The amount derived from boxes is almost identical throughout the country, with the exception of the provincial special hospitals, which get four times as much from this source as any other group.

It has always been supposed that special hospitals receive many fewer legacies than general hospitals. This is not so, however, the proportion being 23·5 per cent. of the whole in the case of general hospitals, and 25·7 per cent. in the case of special hospitals. The general hospitals of London, however, derive no less than 37 per cent. of their income from legacies.

There must be something wrong in the method of distributing the Hospital Saturday Fund in the metropolis, seeing that, whereas the amount given to provincial general hospitals by this organisation represents nearly double the amount received by the special hospitals, in London the reverse is the case, and the Hospital Saturday managers give in proportion 33 per cent. less to the general hospitals than they do to the specials. I commend this fact to the attention of the Council of the Metropolitan Hospital Saturday Fund.

The proportion distributed between the two classes of hospitals by the Hospital Sunday Fund Organisation is pretty uniform throughout the country with the exception of the provinces, where special hospitals get more than the general hospitals. It would seem to be desirable that some steps should be taken throughout England to quicken the interest in Hospital Sunday. There are no Hospital Saturday or Sunday collections in Scotland; and in Ireland, the proportion of the amount raised distributed amongst the general hospitals is identical with the percentage in London.

The admission of paying patients does not seem to make much headway in the metropolitan general hospitals although in the special hospitals 5 per cent of the income is derived from this source. The same holds good of the provincial general hospitals, whilst the provincial special hospitals derive nearly 15 per cent. of their income from patients' payments. This system is also extending amongst the general hospitals of Scotland and about 9 per cent. of the income of the Irish hospitals is derived from this source.

The marked change which has taken place in the Nursing System throughout the country is proved by the fact that ·35 per cent. of the income of all the general hospitals is derived from the payments received for probationary nurses, special hospitals receiving nearly double the amount from that source.

It is satisfactory to note that the sums derived from miscellaneous receipts by general and special hospitals in all parts of the country is almost identical, being 3½ per cent. of the total income.

Simple as these figures may appear, they represent no small expenditure of time and labour extending over a period of more than six weeks.

### HOSPITAL EXPENDITURE.

Having given much consideration to the subject, I have thought it best, as my object is to be helpful rather than critical, not to give the names of the particular institutions in this paper. Anyone who desires to ascertain them will be able to do so on referring to the *Hospital Annual* for 1890 where full details and explanations are given. I purposely confine the figures to the average cost of each bed occupied during the year 1888. This is arrived at by dividing the total average number of beds occupied into the total expenditure, with a deduction of 1s. for each out-patient, and all extraordinary expenses.

### LONDON HOSPITALS.

*Expenditure over £100 per bed occupied.*—Of 56 London Hospitals 21 had an expenditure of over £100 per bed occupied, of which two only are general hospitals, one being a small institution situated in the suburbs.

*Expenditure over £90 per bed occupied.*—There are three such hospitals, *i.e.*, one general, and two special.

*Expenditure over £80 per bed occupied.*—There are six hospitals in this group, of which three are general and three special.

*Expenditure over £70 per bed occupied.*—There are eleven hospitals in this group, of which five are general and six special.

*Expenditure over £60 per bed occupied.*—There are nine institutions in this group, of which three are general and six special.

*Expenditure over £50 per bed occupied.*—There are six in this group, viz., two general and four special.

### PROVINCIAL HOSPITALS.

Of sixty-six provincial hospitals one has an expenditure *over £100 per bed occupied*, one an expenditure *over £90 per bed occupied*, and one an expenditure of *over £80 per bed occupied*; four have an expenditure *over £70 per bed occupied*; all of the foregoing being general hospitals.

*Over £60 per bed occupied.*—There are five hospitals, of which three are general and two special.

*Over £50 per bed occupied.*—There are twenty-nine in this group, all of which are general hospitals.

*Over £40 per bed occupied.*—There are twenty-five hospitals, of which twenty-two are general and three special.

Scotch Hospitals.

There are seventeen Scotch hospitals, of which particulars are forthcoming, but none of these have an expenditure *over £70 per bed occupied.*

*Over £60 per bed occupied.*—There are two, both general hospitals.

*Over £50 per bed occupied.*—There are two, both special hospitals.

*Over £40 per bed occupied.*—There are ten, of which eight are general and two special hospitals.

*Over £30 per bed occupied.*—There are two hospitals, both general.

*Over £20 per bed occupied.*—There is one, a special, hospital.

Irish Hospitals.

I have been enabled to obtain particulars of sixteen institutions, of which one—a county hospital—had an expenditure of *over £100 per bed occupied* in 1887. The authorities have refused particulars this year, on the ground thatthey have long ceased to publish a report.

*Over £80 per bed occupied.*—There is one special hospital.

*Over £50 per bed occupied.*—There are four hospitals, two of which are general and two special.

*Over £40 per bed occupied.*—There are four institutions, of which three are general and one special.

*Over £30 per bed occupied.*—There are three institutions, of which one is a general and two are special hospitals.

*Over £20 per bed occupied.*—There are three general hospitals.

We have thus information concerning one hundred and fifty-five general and special hospitals in England and Wales, Scotland, and Ireland, and it will be admitted by everybody that the figures afford much food for reflection, that the difference between the cost of an occupied bed on the highest average, which in the case of one special hospital for lying-in cases amounts to no less a sum than £199 4s., and that of the lowest average cost per occupied bed, represented by a children's hospital in Scotland, where it amounts to only £27 2s., is as remarkable as it is unaccountable, at the first blush at any rate. It is fair to add, however, that in the case of the lying-in hospital there were one thousand three hundred and fifty-four out-patients, which, if estimated to cost 10s. per patient, would reduce the cost per bed to about £161, making another special hospital, one for diseases of the eye, the highest on the list, as the cost per occupied bed was in this case £178 12s. 6d. It may be mentioned that one of the London children's hospitals has an expenditure per occupied bed of £124 18s. 9d., and that nearly every class of hospital is represented on the London list, where the cost per occupied bed exceeds £100 each.

*(To be continued.)*

